# Rationally repurposed nitroxoline inhibits preclinical models of Epstein–Barr virus-associated lymphoproliferation

**DOI:** 10.1038/s41429-021-00433-2

**Published:** 2021-06-23

**Authors:** Maite Ibáñez de Garayo, Wendi Liu, Nicole C. Rondeau, Christopher B. Damoci, JJ L. Miranda

**Affiliations:** 1 Department of Biology, Barnard College, Columbia University, New York, NY 10027 USA; 2 Herbert Irving Comprehensive Cancer Center, Columbia University Irving Medical Center, New York, NY 10032 USA; 3 Present Address: Department of Pathology and Laboratory Medicine, Weill Cornell Medical College, New York, NY 10065 USA

**Keywords:** Chemotherapy, Haematological cancer, Drug discovery, Antibiotics, Epigenetics

## Abstract

Repurposing of currently used drugs for new indications benefits from known experience with those agents. Rational repurposing can be achieved when newly uncovered molecular activities are leveraged against diseases that utilize those mechanisms. Nitroxoline is an antibiotic with metal-chelating activity used to treat urinary tract infections. This small molecule also inhibits the function of bromodomain and extraterminal (BET) proteins that regulate oncogene expression in cancer. Lymphoproliferation driven by the Epstein–Barr virus (EBV) depends on these same proteins. We therefore tested the efficacy of nitroxoline against cell culture and small animal models of EBV-associated lymphoproliferation. Nitroxoline indeed reduces cell and tumor growth. Nitroxoline also acts faster than the prototype BET inhibitor JQ1. We suggest that this rational repurposing may hold translational promise.

## Introduction

Repurposing of known drugs, also referred to as repositioning, for cancer treatment offers substantial benefits over development of new therapeutic compounds. For example, an established track record of clinical use allows for swifter indication as standard of care because safety profiles already benefit from a wealth of prior experience. While repositioning often occurs through serendipity, rational repurposing may arise when new molecular properties or mechanisms of actions are discovered. We were encouraged by newfound regulation of epigenetics to explore preclinical validation of the small molecule antibiotic nitroxoline for the treatment of lymphoproliferation driven by the Epstein–Barr virus (EBV).

Nitroxoline is an antibiotic prescribed for decades in Europe to treat urinary tract infections [1]. Antibacterial activity can be attributed to the compound’s ability to chelate metal ions [2, 3] and dispel biofilms [4]. Years of use has led to a good understanding of nitroxoline pharmacokinetics [5]. A high-throughput screen discovered inhibition of human cell proliferation and subsequent work in mouse models of cancer revealed related efficacy against human urothelial carcinoma and invasive ductal carcinoma xenografts [6]. Potential antitumor properties include the ability to inhibit methionine aminopeptidase [6] and cathepsin B [7]. Efforts at repurposing nitroxoline have uncovered molecular mechanisms of anticancer activity [8, 9] and succeeded against human xenografts in mouse models of clear cell renal cell carcinoma [10], multiple myeloma [11], and glioma [12]. A more recent high-throughput screen identified nitroxoline as a competitive inhibitor of the bromodomain and extraterminal (BET) family of chromatin regulators [13]. This new molecular activity suggests repurposing against cancers that leverage these particular epigenetic proteins for proliferation.

EBV immortalizes B cells by co-opting BET protein function. Epigenetic deregulation during cancer results in the formation of super-enhancers dependent on BET proteins such as BRD4 to drive the expression of oncogenes [14]. Infection by EBV can similarly assemble super-enhancers consisting of viral proteins and BRD4 [15]. These viral proteins are expressed in what is termed type III EBV latency, a stereotyped transcription program found in most cases of EBV-associated posttransplant lymphoproliferative disease (PTLD) [16]. The prototype BET inhibitor JQ1 reduces growth of EBV-associated lymphoproliferation in cell culture [15, 17]; we also further demonstrated efficacy in a mouse model [17]. Unfortunately, no BET inhibitors are approved for clinical use [18]. We therefore hoped to help validate the repurposing of the antibiotic nitroxoline against EBV-associated lymphoproliferation.

Lymphoblastoid cell lines (LCLs) serve as a model of EBV-associated lymphoproliferation similar to that observed in PTLD. We grew the EBV-positive GM12878 and 721 LCLs in culture [17, 19]. GM12878 is a suspension cell line without translocations that was derived from B lymphocytes transformed by EBV and is frequently examined in epigenetic studies [20]; 721 was derived independently but similarly [21]. The type III viral latency transcription programs of GM12878 and 721 LCLs [19] match those observed in most cases of EBV-associated PTLD [16]. We treated cells with nitroxoline (Sigma-Aldrich, St. Louis, MO, USA), JQ1 (Selleck Chemicals, Houston, TX, USA), or a DMSO vehicle control as previously described for JQ1 [17]. Proliferation was measured by counting cells. Viability was measured with trypan blue, which detects membrane integrity. Metabolic activity was measured with PrestoBlue Cell Viability Reagent (Thermo Fisher Scientific, Waltham, MA, USA), which detects reducing power. Cell counts and trypan blue exclusion were measured on a Countess II FL Automated Cell Counter (Thermo Fisher Scientific, Waltham, MA, USA) after seeding GM12878 cells at a density 0.3 × 10^6^ cells ml^−1^ and culturing for 3 days. PrestoBlue Cell Viability Reagent was measured on a Spark multimode microplate reader (Tecan, Männedorf, Switzerland) at an absorbance wavelength of 570 nm after seeding GM12878 or 721 cells at a density 0.1 × 10^6^ cells ml^−1^ and culturing for up to 3 days. Statistical comparisons were made with paired Student’s *t* tests.

**Fig. 1:**
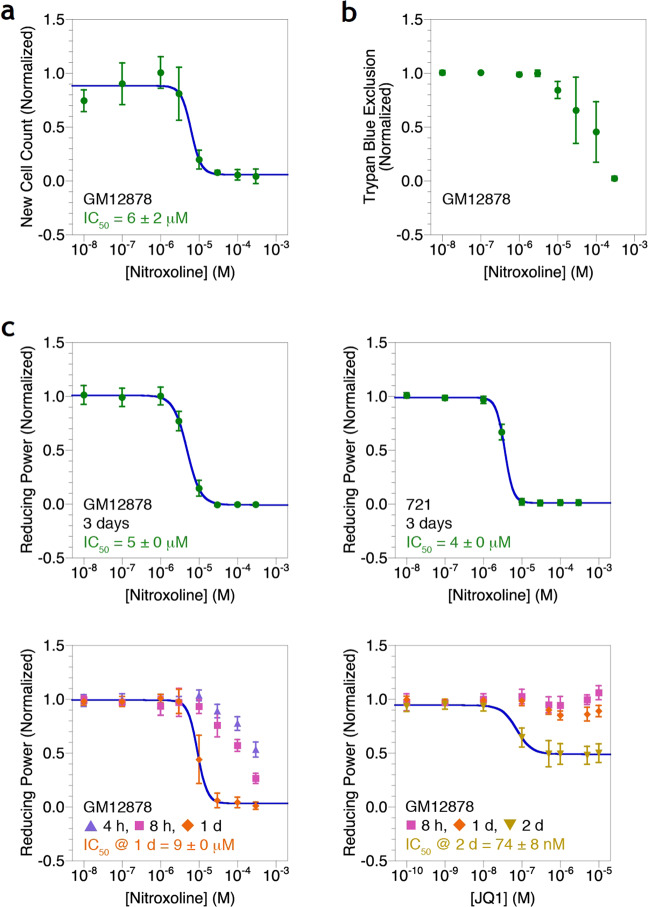
Nitroxoline reduces proliferation of EBV-immortalized LCLs in cell culture. **a** Growth of the GM12878 line in cell culture after 3 days of nitroxoline treatment. Expansion is measured as cell density increase normalized to the vehicle control. Error bars represent the standard deviation of *n* = 4 replicates. **b** Viability of the GM12878 line in cell culture after 3 days of nitroxoline treatment. Membrane integrity is measured as trypan blue exclusion normalized to the vehicle control. Error bars represent the standard deviation of *n* = 4 replicates. **c** Metabolic activity of the GM12878 and 721 lines in cell culture after up to 3 days of nitroxoline or JQ1 treatment. Reducing power is measured as PrestoBlue Cell Viability Reagent absorbance normalized to the vehicle control. Error bars represent the standard deviation of *n* = 4 replicates

Nitroxoline reduces growth of EBV-associated lymphoproliferation in cell culture. Nitroxoline reduces growth of the GM12878 LCL by decreasing the proliferation of new cells (Fig. 1a), decreasing viability (Fig. 1b), and decreasing metabolic activity (Fig. 1c). Viability is perturbed at a higher concentration than proliferation and metabolic activity because membrane integrity is usually impacted at a later stage of cell death than cell count and reducing power. To demonstrate generality, nitroxoline also decreases metabolic activity of the 721 LCL (Fig. 1c). These effects are observed at low μM concentrations achievable in humans [22, 23]. We previously demonstrated that the prototype BET inhibitor JQ1 reduces growth of GM12878 cells both in cell culture and in a mouse model [17]. To compare nitroxoline with JQ1, we measured metabolic activity of GM12878 cells over a time course (Fig. 1c). Nitroxoline reduces metabolic activity much more potently than JQ1 under these conditions. After 8 h and 1 day, JQ1 shows very little effect on reducing power, only achieving substantial growth inhibition after 2 days. Nitroxoline shows moderate effects after 8 h and even as early as 4 h, achieving substantial growth inhibition after 1 day. Nitroxoline therefore acts with faster kinetics than JQ1. Encouraged by these results in cell culture, we then tested the efficacy of nitroxoline in a small animal model of cancer.

We assessed efficacy in a mouse model with LCL xenografts as previously described for JQ1 [17]. Nitroxoline was suspended in soybean oil for dosing. Weight was also measured in addition to tumor size. Statistical comparisons were made with Mann–Whitney–Wilcoxon rank sum tests.

**Fig. 2:**
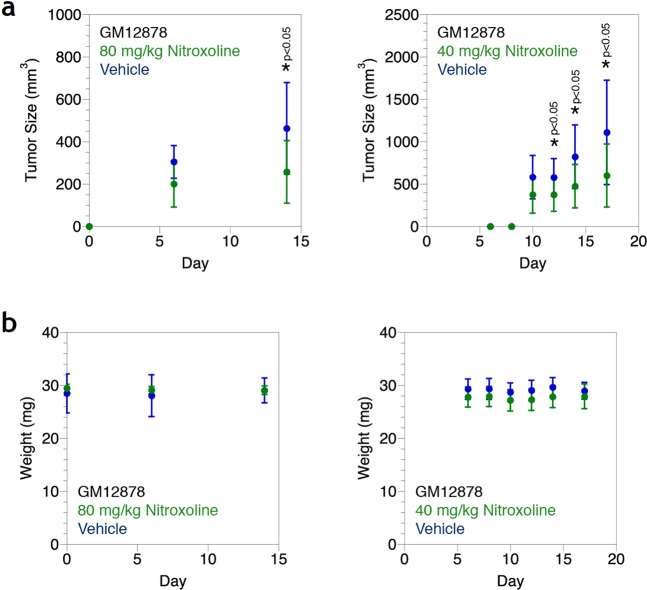
Nitroxoline reduces proliferation of EBV-immortalized LCLs in an animal model. **a** Expansion of engrafted GM12878 cells in NSG mice treated with nitroxoline. Expansion is measured as tumor volume. Error bars represent the standard deviation of *n* = 8 mice. **b** Health of NSG mice treated with nitroxoline. Health is measured as weight. Error bars represent the standard deviation of *n* = 8 mice

Nitroxoline reduces growth of EBV-associated lymphoproliferation in a small animal model. A dose of 80 mg kg^−1^ intraparietal nitroxoline daily reduces tumor sizes after ~2 weeks (Fig. 2a). Tumor volume decreases by ~40%. No toxicity is observed during the time course as weight, a gross indicator of health, is not affected by nitroxoline treatment (Fig. 2b). A lower dose of 40 mg kg^−1^ intraparietal nitroxoline daily also reduces tumor sizes by ~40–50% after ~2 weeks (Fig. 2a) without weight loss (Fig. 2b).

We cautiously note that we did observe statistically insignificant mortality in the nitroxoline group with extended treatment. Prolonged dosing beyond the ~2-week time frame of tumor size reduction without mortality (Fig. 2a) resulted in loss of mice. With 80 mg kg^−1^ nitroxoline, two out of eight mice did not survive, one lost on day 15 and another on day 21. With 40 mg kg^−1^ nitroxoline, one out of eight mice did not survive, lost on day 19. These differences in survival were not significant according to a Fisher exact test. The cause of mortality is unclear given the lack of weight loss up to (Fig. 2b) and inclusive of those time points (data not shown). No abnormal findings were observed during animal care (data not shown). We find it difficult to speculate with confidence that either BET inhibition or metal chelation is related to mouse death. Technical error during repeated intraperitoneal dosing is possible. Other experiments treating mice with nitroxoline at higher doses for longer times also showed no increased mortality compared to vehicle [10]. Additional investigation into the long-term safety profile of nitroxoline may nonetheless be necessary.

Even though work remains, PTLD treatment strategies could benefit from additional options [24, 25]. A rationally repurposed drug may allow safe and rapid translation into the clinic. Nitroxoline indeed reduces the growth of EBV-associated lymphoproliferation in both cell culture and a mouse model. We contend that our preclinical studies support usage of the antibiotic nitroxoline against EBV-associated malignancies as seen in PTLD.
